# Risk factors for development and progression of chronic kidney disease in elderly Chinese patients with diabetes: who has been forgotten

**DOI:** 10.1080/0886022X.2023.2294152

**Published:** 2023-12-18

**Authors:** Yu Zhao, Yanan Wang, Zhilong Dong

**Affiliations:** a Department of the Second Hospital & Clinical Medical School, Lanzhou University, Lanzhou, PR China; b Department of Medicine, Northwest University for Nationalities, Lanzhou, PR China; c Department of Urology, The Second Hospital of Lanzhou University, Lanzhou, PR China

Dear Editor,

The paper titled ‘Serum uric acid is associated with chronic kidney disease in elderly Chinese patients with diabetes’ by Zhou et al. [1] aroused us great interest. This community-based cohort study included 39,039 elderly diabetic patients without chronic kidney disease (CKD) for the 2-year follow-up to analyze the relationship between hyperuricemia or elevated serum uric acid (SUA) levels within the normal range and the new-onset CKD. Multivariate Cox regression analysis showed that hyperuricemia for new-onset CKD were 1.925 (1.724–2.150) and 1.676 (1.520–1.848) for males and females; drinking for new-onset CKD were 0.779 (0.684–0.887) and 0.605 (0.376–0.972), respectively, independent of age, sex, diabetes duration, obesity, hypertension, systolic blood pressure, diastolic blood pressure, smoking, drinking, dyslipidemia, triglyceride, total cholesterol, high-density lipoprotein cholesterol, low-density lipoprotein cholesterol, and fasting plasma glucose. I pay special attention to the influencing factors of the research because it caused us some confusion.

**Figure 1.**
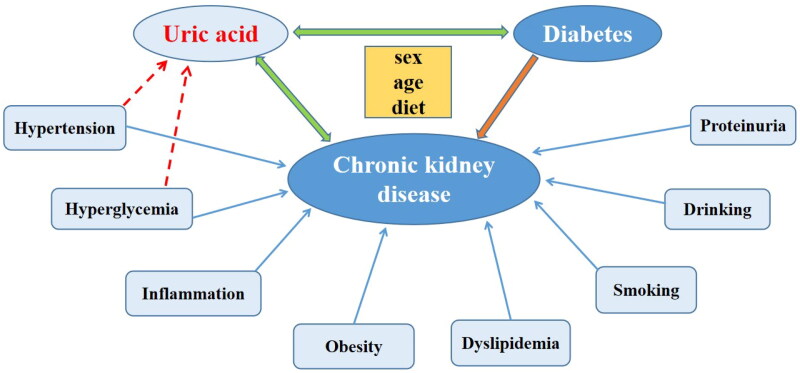
Interaction of uric acid, diabetes and CKD, and major risk factors for CKD. (sex, age and diet are common influence factors; the solid blue arrow indicates the risk factors for CKD; the red dashed arrow indicates a correlation with uric acid. *Note*. There is a risk of incomplete summary.

As shown in Figure 1, uric acid, diabetes and CKD have complex relationships that influence each other. Uric acid and diabetes are important risk factors for the development and progression of CKD, but proteinuria and diet were not included in the original article by Zhou et al. [1]. Albuminuria is useful for the early screening and diagnosis of kidney impairment, especially in individuals with pre-diabetes or type 2 diabetes, which is the leading cause of CKD and end-stage kidney disease [2]. A meta-analysis demonstrated that decreasing albuminuria was associated with a reduced risk of CKD progression [3].

Second, diet. A nationwide representative study including 66,427 Chinese adults aged 18 years and above were conducted to explore the association of dietary approaches to stop hypertension (DASH) diet with SUA levels and the odds of hyperuricemia. The study established that a higher DASH score was associated with lower SUA levels (*β* = −0.11, 95% CI −0.12, −0.1, *p* < 0.001) and odds of hyperuricemia (OR = 0.85, 95% CI 0.83–0.87, *p* < 0.001) in Model 2 [4]. Meta-analysis revealed dietary association with hyperuricemia risk: red meat: OR 1.24 (95% CI 1.01–1.48); seafoods: OR 1.47 (95% CI 1.16–1.86); alcohol:OR 2.06 (95% CI 1.60–2.67); fructose: OR 1.85 (95% CI 1.66–2.07); diary products: OR 0.5 (95% CI 0.37–0.66); soy foods: OR 0.7 (95% CI 0.56–0.88); high-purine vegetables ingestion: OR 1.10 (95%CI 0.88–1.39, *p* = 0.39); coffee: OR 0.76 in men (95% CI 0.55–1.06, *p* = 0.11), OR 1.58 in women (95% CI 1.16–2.16) [5]. An umbrella review by syntheses evidence on diet interventions and dietary factors in the prevention of type 2 diabetes showed that the DASH diet had protective effects on type 2 diabetes, with the pooled RRs ranging from 0.73 (95% CI 0.65–0.83) to 0.79 (95% CI 0.66–0.95) [6].

**Figure 2.**
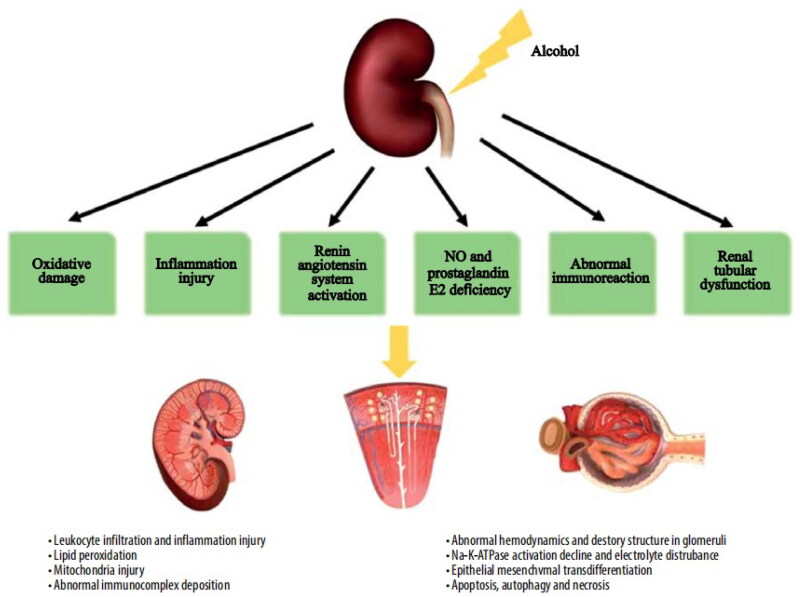
The possible mechanism of alcohol-induced renal injury (from Ref. [7]).

Once again, drinking is a complex social activity. A review revealed that alcohol consumption can be a ‘double-edged sword’ for CKD patients, as age, sex, primary disease, initial eGFR, genetic, individual differences, drinking pattern, integral dose of alcohol consumption, differences in alcohol beverages, and various concomitant factors can affect the prognosis of patients with CKD and interfere with the effects of alcohol on the kidneys, also summarized the possible mechanism of alcohol-induced renal injury (Figure 2) [7]. In addition, neither the CKD-EPI nor the MDRD Study equation is optimal for all populations and GFR ranges. CKD-EPI equation might be a superior surrogate marker of GFR in patients with normoalbuminuria and hyperfiltration and could be used as a screening tool for early renal impairment in diabetes. The back propagation neural network tool is more accurate than the currently available creatinine-based GFR estimation equations in older populations and could be recommended for routine clinical use [8].

Finally, I found that the sum of the number of diabetes with or without hyperuricemia was inconsistent with the total number of included patients in Figure 1, and the 38th reference was incomplete due to the absence of the author’s message [1]. Therefore, the accuracy of the details has yet to be further verified by the author.
